# Stable *establishment* of wMel *Wolbachia* in *Aedes aegypti* populations in Yogyakarta, Indonesia

**DOI:** 10.1371/journal.pntd.0008157

**Published:** 2020-04-17

**Authors:** Warsito Tantowijoyo, Bekti Andari, Eggi Arguni, Nida Budiwati, Indah Nurhayati, Iva Fitriana, Inggrid Ernesia, Edwin W. Daniwijaya, Endah Supriyati, Dedik H. Yusdiana, Munasdi Victorius, Dwi S. Wardana, Hilmi Ardiansyah, Riris Andono Ahmad, Peter A. Ryan, Cameron P. Simmons, Ary A. Hoffmann, Edwige Rancès, Andrew P. Turley, Petrina Johnson, Adi Utarini, Scott L. O’Neill

**Affiliations:** 1 World Mosquito Program Yogyakarta, Centre for Tropical Medicine, Faculty of Medicine, Public Health and Nursing, Universitas Gadjah Mada, Yogyakarta, Indonesia; 2 Department of Pediatrics, Faculty of Medicine, Public Health and Nursing, Universitas Gadjah Mada, Yogyakarta, Indonesia; 3 Department of Biostatistics, Epidemiology and Population Health, Faculty of Medicine, Public Health and Nursing, Universitas Gadjah Mada, Yogyakarta, Indonesia; 4 Institute of Vector-Borne Disease, Monash University, Clayton, Melbourne, Victoria, Australia; 5 University of Melbourne, Melbourne, Victoria, Australia; 6 Department of Health Policy and Management, Faculty of Medicine, Public Health and Nursing, Universitas Gadjah Mada, Yogyakarta, Indonesia; University of California, Davis, UNITED STATES

## Abstract

The successful establishment of the *w*Mel strain of *Wolbachia* for the control of arbovirus transmission by *Aedes aegypti* has been proposed and is being implemented in a number of countries. Here we describe the successful establishment of the *w*Mel strain of *Wolbachia* in four sites in Yogyakarta, Indonesia. We demonstrate that *Wolbachia* can be successfully introgressed after transient releases of *w*Mel-infected eggs or adult mosquitoes. We demonstrate that the approach is acceptable to communities and that *Wolbachia* maintains itself in the mosquito population once deployed. Finally, our data show that spreading rates of *Wolbachia* in the Indonesian setting are slow which may reflect more limited dispersal of *Aedes aegypti* than seen in other sites such as Cairns, Australia.

## Author summary

We show that the wMel strain of *Wolbachia* can be deployed successfully into mosquito populations in an area of intense dengue transmission in Yogyakarta, Indonesia. Deployment was achieved through either the release of eggs or adult mosquitoes with full community support. This represents the successful first step toward a large trial to evaluate the use of *Wolbachia* in Indonesia to disrupt transmission of arboviruses such as dengue, Zika and chikungunya.

## Introduction

There is a substantial body of evidence that *Aedes aegypti* mosquitoes infected with *Wolbachia* have lower transmission potential for human arboviruses like dengue, Zika and chikungunya [1–5]. These observations form the basis for a new approach to arbovirus control, namely the release of mosquitoes containing *Wolbachia* with the goal of establishing *Wolbachia* infection in wild mosquito vector populations and so interrupting local virus transmission from *Aedes* mosquitoes to humans. This approach has been undergoing open field testing in a number of countries by the World Mosquito Program (WMP) (formerly known as the Eliminate Dengue Program) since 2011 (www.worldmosquitoprogram.org). This approach differs fundamentally from that of other groups that are testing the use of *Wolbachia* to suppress mosquito populations through release of *Wolbachia* infected males [6]. This latter approach does not result in *Wolbachia* establishing in the wild mosquito population. It also requires higher mosquito release numbers and ongoing releases in contrast to the WMP approach which only undertakes releases to establish *Wolbachia* and with the intention that it persists in the mosquito population through the action of cytoplasmic incompatibility (CI). This removes the need for additional releases and provides for a more cost effective and sustainable approach to disease control.

The first open releases of *Wolbachia* infected *Ae*. *aegypti* were undertaken in northern Australia in isolated pilot locations [7]. This initial work showed that the *w*Mel strain could establish and maintain itself after relatively small introductory releases. Long-term monitoring of these sites shows that the *w*Mel strain of *Wolbachia* is able to maintain itself stably in these mosquito populations at frequencies typically above 90% without reapplication [8]. Moreover, when wild mosquitoes are sampled from the field years after initial releases were completed the mosquitoes remain less susceptible to dengue virus infection, indicating phenotypic stability [9]. Subsequent releases around the Cairns area of Australia have shown that *Wolbachia* can also be introduced into pilot areas in contiguous urban habitat and this has allowed for spreading speeds to be estimated for the *w*Mel *Wolbachia* strain [10].

In this paper, we describe the first successful deployments of the *w*Mel strain of *Wolbachia* in the province of Yogyakarta, Indonesia. Yogyakarta, like most of Indonesia [11, 12], is dengue endemic [13]. Mosquito releases were undertaken into four communities on the outskirts of the city area of Yogyakarta in preparation for a future large Cluster Randomised Trial to test the epidemiological impact on dengue in the city. These releases allowed us to demonstrate the feasibility of establishing *Wolbachia* in a disease endemic setting, the community acceptability of the approach, as well as compare different release modalities and the long-term durability of *Wolbachia* establishment.

## Methods

### Ethics statement

Approval for human blood-feeding of mosquito colonies and field release of mosquitoes was provided by the Medical-Health Research Ethical Committee, Faculty of Medicine, Universitas Gadjah Mada Ethics Committee, approvals KEI0611112011 and KE/FK/818/EC respectively. Individual written consent to be involved in the project was obtained in Nogotirto and Kronggahan. Approximately 95% of adults aged 17 or above provided consent. For the villages of Jomblangan and Singosaren this was changed to community consent where each Rukun Tetangga (the smallest formal community administrative unit within Indonesia–around 40–50 households) elected leader (16 in total) provided consent for their respective communities. All 16 leaders consented to their communities to be involved with the project.

### Release sites

Four release sites were chosen based on three selection criteria. First, they were located outside of Yogyakarta city and were isolated from other residential areas with at least 80m separating houses in the potential release site from any neighbouring houses. Second, they were sites where *Ae*. *aegypti* was commonly found and third, they were areas that were considered willing to participate in the study. S1 Fig shows a map of the chosen sites, Kronggahan, Nogotirto, Jomblangan and Singosaren, in relation to the city of Yogyakarta and S1 Table shows descriptive information for each site.

### Mosquito rearing and release strain preparation

Wild-type Yogyakarta mosquitoes were collected periodically using 400 ovitraps (with flannel cloth strips for oviposition) placed in the four release sites, Kronggahan, Nogotirto, Jomblangan and Singosaren. Ovitraps were set for 1 week and strips were then returned to the insectary, kept damp for 2 days, and then air dried for 1 day. Eggs were hatched, reared to the 3-4rd instar larvae, and *Ae*. *aegypti* were retained and fed sheep blood with a Hemotek membrane feeder. Resulting eggs (F1) were harvested weekly for 4–8 gonotrophic cycles. Eggs were kept damp for 2 days, dried for 1 day, and stored in a humidified sealed plastic container and kept at room temperature (25°C ± 2°).

Wild-type F1 eggs were hatched and then used for either backcrossing or outcrossing of the release strain as described below. The release strain for Kronggahan and Nogotirto was generated by backcrossing F1 wild-type *Ae*. *aegypti* males (ovitrapped and pooled from Kronggahan, Nogotirto, Jomblangan and Singosaren) with females from a colony of mosquitoes from Australia containing the *w*Mel *Wolbachia* strain [2] for five generations. This was done to introgress the local genetic profile (including locally selected insecticide resistance genes) into the *w*Mel-infected *Ae*. *aegypti* founder colony from Australia.

At the completion of backcrossing the release strain was maintained as a colony in which 10–20% of the males for each generation were wild-type F1 to ensure the colony was further outbred. For releases into Jomblangan and Singosaren the release strain was generated by taking the first release strain and then backcrossing it for one generation with wild-type males collected from these sites and then further outcrossed by adding 10–20% wild-type males to each successive generation.

Mosquitoes were reared in an insectary maintained at ambient temperature (26–29°C) in 3.4 litre buckets filled with 1.2 litres of water and fed TetraMin fishfood (Tetra, Germany). Approximately 400 larvae were reared in each bucket. The 1^st-^2^nd^ instar larvae (or 24 hours after hatching) were fed a ¼ tea spoon of TetraMin, then 3^rd^ and 4^th^ instars (usually 4 and 5 days after hatching) were fed another ¼ tea spoon of TetraMin. Adults were maintained in 30 cm^3^ cages and bloodfeeding was done with human volunteers as per previous protocols [7, 14]. All healthy volunteer bloodfeeders were afebrile and free of clinical signs or symptoms of an arbovirus infection both at the time of bloodfeeding and for 3 days thereafter.

### Quality assurance

Before field releases, and every 6 months, the colony underwent checks for a number of fitness measures including *Wolbachia* maternal transmission rate, fecundity, hatch rate and wing length. In addition, weekly testing of colony material was undertaken prior to release to ensure that *Wolbachia* infection prevalence was >96%. To this end, 150 early instar larvae were taken from the colony each week prior to release and underwent PCR testing for *Wolbachia* (see below). In all of the release experiments the *Wolbachia* frequency in the release material was greater than 98%. Additionally, to ensure that released adults were fit, a sample of 10 release cups were divided into two groups and one group was kept in the insectary and the other group went out to the field for release but was held and returned to the insectary after the release run. Adult survival was then measured in both groups of cups for a subsequent 7 days to ensure survivorship exceeded 90%. For egg releases this measure was substituted with egg hatch experiments where adult survival was replaced with hatchability of eggs intended for the field. Five egg batches from the insectary were hatched and another five egg batches went out on the release run and were returned to the insectary before hatching to ensure that hatch rates were greater than 90%.

### Community engagement and consent

Our objective through our community engagement activities was to obtain continuous community support for project activities. This was facilitated by an approach that relied heavily on face to face engagement with individuals to build trust and positive relationships. Considering that this project covered a population of around 9,000 community members we utilized a number of engagement activities to effectively engage with residents with a similar overarching framework that had been used successfully previously [14, 15]. These included:

1. The use of baseline surveys to assess public awareness and acceptance of the release of *Wolbachia* mosquitoes.
2. Establishment of community reference groups in each site to oversee the engagement activities of the project. These groups were formed within the existing community representative council of each village (LMPD).
3. Working closely with Puskesmas (public health centres) staff to disseminate project information.
4. Utilising existing community meeting structures to present to community members and hear and respond to concerns. From Jan 2012 to June 2013, 296 such meetings were attended. In addition, using other existing community events to inform people about the project.
5. Establishing field offices in each site to have a recognizable base in each community where we could easily be contacted.
6. Establishing an issues management system to respond to community enquiries. Community was able to contact project through direct contact with staff, a telephone hotline, email or text messages.
7. Recruiting a group of people that served as community champions for the project. These people were respected individuals in the community we supplied with deeper knowledge about the program and that helped increase awareness about the project and mediate if needed on any community concerns. Included in these people were the local women’s health collaborators that were able to ensure that our program was aligned with government policy around mosquito breeding site control.

### Adult Releases in Nogotirto and Kronggahan (Sleman District)

**Fig 1:**
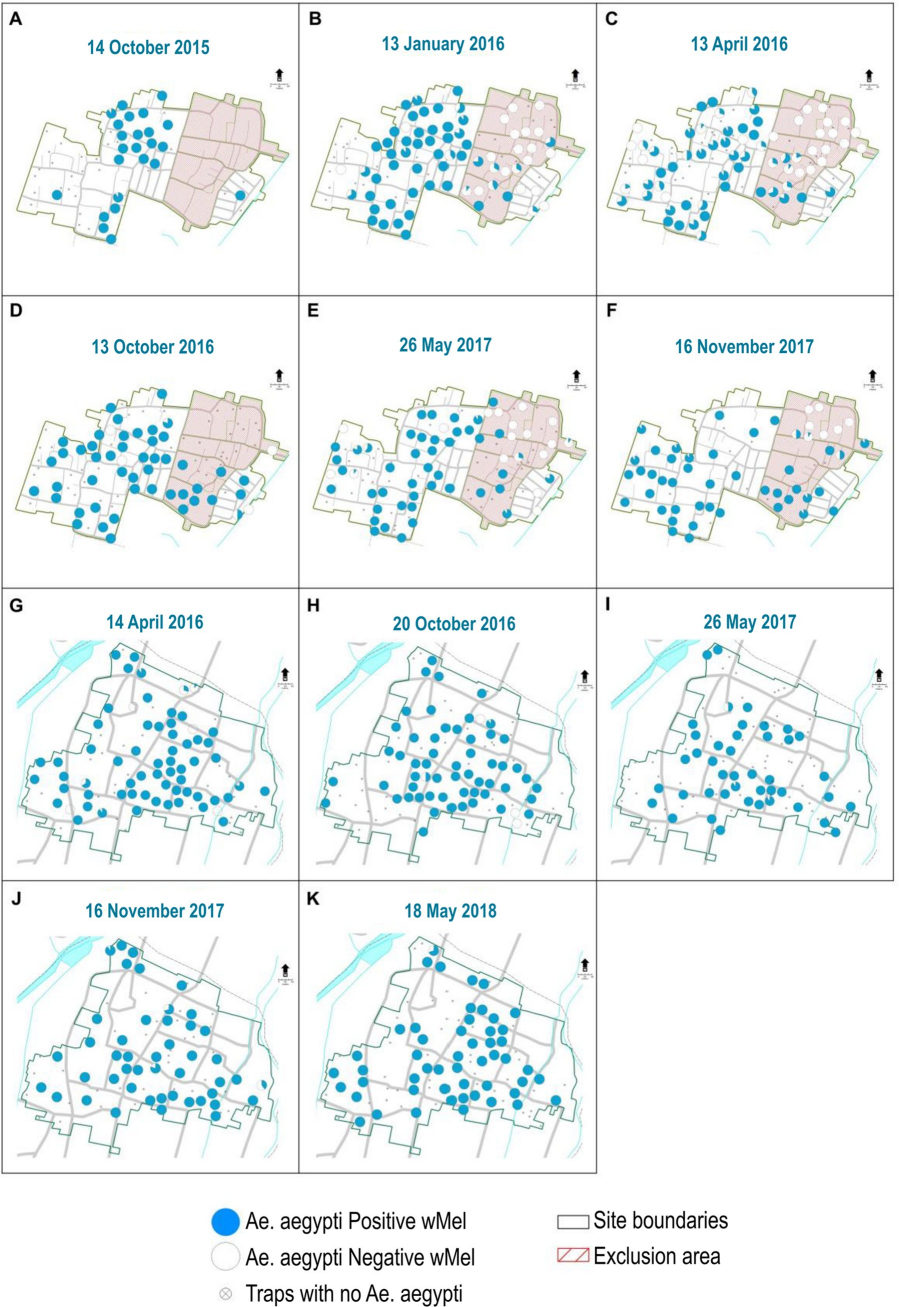
Ovitrap monitoring of *Wolbachia* frequency in Nogotirto (A, B, C, D, E, F) highlighting the slow spatial spread of *w*Mel *Wolbachia* into the exclusion area where releases were not undertaken and Kronggahan (G, H, I, J, K). Pie graphs show location of where ovitraps were set and the amount of blue shading indicates frequency at each trap from a maximum sample of 10 individuals. Maps supplied by Indonesian Geospatial Information Agency and processed with ArcGIS.

Adult mosquito releases were undertaken in the communities of Nogotirto and Kronggahan beginning in Jan 2014. In both communities, a 25m^2^ grid was used to assign release points with a single release point assigned to each 25m^2^ grid. However, in a given week releases were only undertaken over 50% of the release points with an even spacing across both sites and then in alternate weeks the other release points were used. At each release point a cup of 40 adult male and female mosquitoes with an approximately even sex ratio was released. Adults were 4–5 days old when released and had fed on sugar or honey solution *ad libutum*. Releases were undertaken over a total of 20 weeks. In the community of Nogotirto there was opposition from some community leaders to undertake releases in an area of the site (Fig 1). This desire was respected and no mosquitoes were released in this area. Nonetheless we established a monitoring network throughout the exclusion area to measure spread of *Wolbachia* from the release area. In addition, across all sites there were a small number of community members that did not want mosquitoes released near their houses. This led to a negotiated 25-50m diameter exclusion area for releases centred on the household not consenting to release.

### MRC Releases in Jomblangan and Singosaren (Bantul District)

In the communities of Jomblangan and Singosaren, releases were performed from November 2014-May 2015. As above, a 25m^2^ grid-based release approach was employed in both communities, but the release material consisted of eggs in a mosquito release container (MRC). One mosquito release container (MRC) with 80–120 eggs was placed in a position shaded from direct sun and rain at a residential property within each grid square. MRC’s were 2 litre plastic buckets with 8 holes of 0.7 cm diameter to allow adult mosquitoes to escape. MRC’s were filled with ~1 liter of water and 5–6 pellets of fish food (Tropical Carnivore, Tropical Tadeusz Ogrodnik, Poland). MRCs were serviced (old water discarded and clean water, new eggs and fish food added) after 9 days and where possible moved to a neighbouring property within the 25m^2^ grid square. This was performed for 12 rounds. Quality assurance was performed by counting pupal skin, dead larvae and pupae and adults in a randomly selected third of buckets. Based on this surveillance of MRCs the mean number of released adults per MRC per cycle was 63 (min = 45, max = 81) in Jomblangan and 62 (min = 28, max = 90) in Singosaren.

### BGS surveillance for Wolbachia prevalence

Biogents Sentinel adult mosquito traps (BGS traps) were spatially distributed in line with local administrative units, with 3–5 BGS traps placed per hamlet (~40 houses). Traps without attractants were placed indoors in residential properties in the corner of the living room. All traps were connected to mains electricity supply with battery back-up and run continuously. Homeowners were compensated for additional electricity costs. Mosquito trapping commenced prior to *Wolbachia* deployment to estimate the population size. BGS traps were serviced every week and collected *Ae*. *aegypti* identified based on morphological characteristics. *Ae*. *aegypti* were transferred to 85% ethanol and then tested for the presence of *Wolbachia*.

### Ovitrap monitoring

Ovitrapping was conducted as a pragmatic method to measure the long-term establishment of *Wolbachia*. Across each study location, ~100 ovitraps (spatially evenly distributed) were set up indoors in residential properties at a ratio of one trap per property and spatially evenly distributed. Ovitraps were plastic buckets 13cm in diameter and 12 cm high. Each bucket was filled with approximately 750 ml of water, with 6 pellets of rabbit food (NOVA, Perfect Companion Group Co., Thailand) as bait to attract females. Two cotton flannel strips were added to the sides of the buckest sides to support oviposition. After 1 week in the field, traps were collected and flannel strips with mosquito eggs dried by evaporating at room temperature for 3 days. Eggs were then hatched and reared to adults in the laboratory and from each ovitrap 10 three-five day old *Ae*. *aegypti* mosquitoes (random selection of males/females) were tested for the presence of *Wolbachia* infection by PCR. From ovitrapping, The *Wolbachia* prevalence for each site was then estimated as total number of *Ae*. *aegypti* that tested positive for Wolbachia divided by the total number tested.

### Wolbachia diagnostics

DNA was extracted from adult mosquitoes and tested for the presence of *Wolbachia* using a TaqMan PCR for *Wolbachia* infection as previously described [7, 14].

## Results and discussion

**Fig 2:**
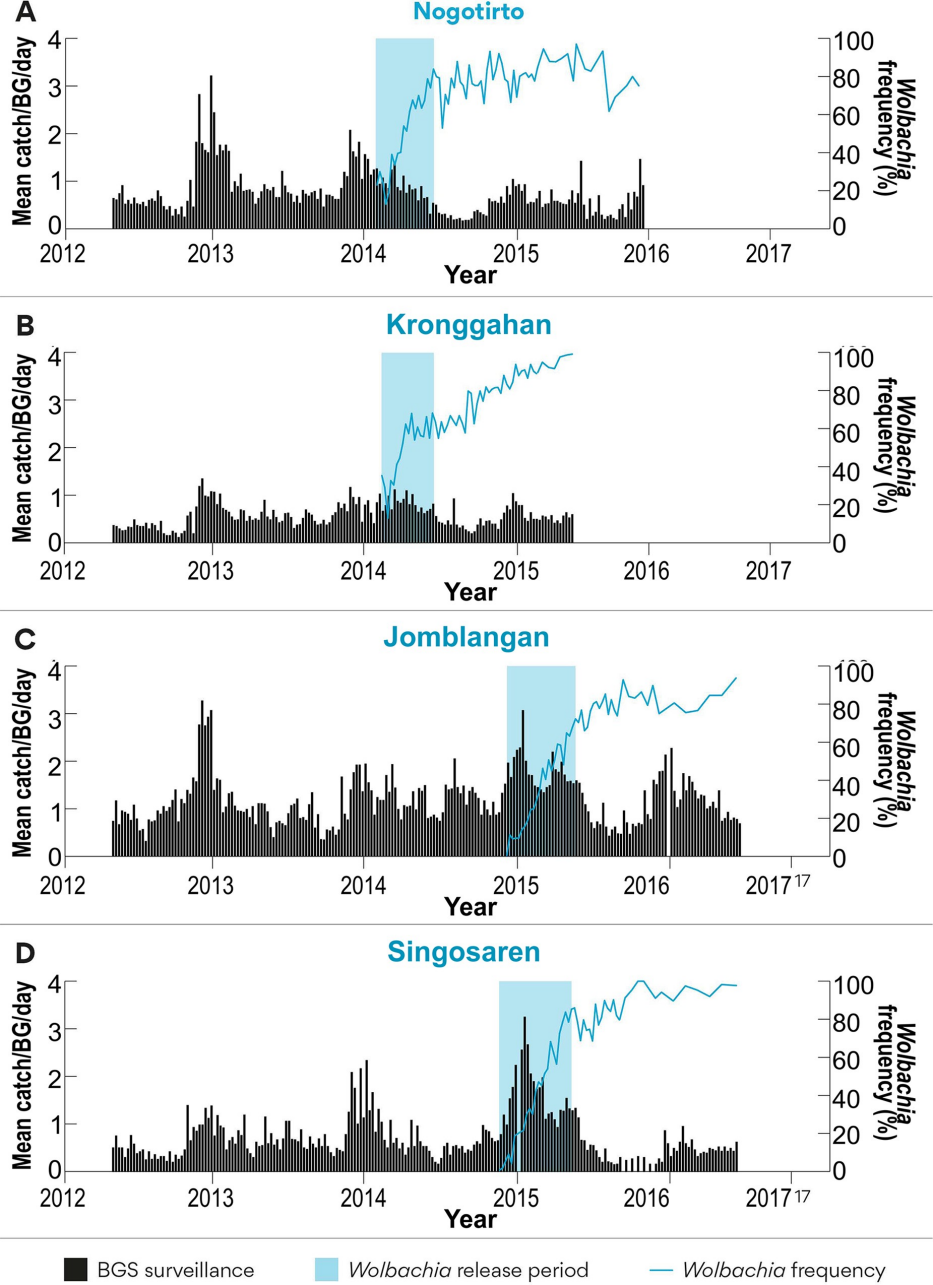
*w*Mel establishment in each of the four sites. Nogotirto and Kronggahan were used as adult release sites and Jomblangan and Singosaren were used as egg release sites. Each panel shows background *Ae*. *aegypti* population as measured by BGS trap sampling. Blue shading indicates the period of mosquito release and the blue line shows the *w*Mel frequency obtained from mosquitoes sampled in BGS traps.

Mosquitoes containing *w*Mel were released as adults in Nogotirto and Kronggahan (both Sleman District) in 2014 and then as eggs in an additional two sites, Jomblangan and Singosaren (both Bantul District) in 2015. In Sleman District 20 rounds (20 weeks) of adult releases led to *w*Mel being established successfully (Fig 2). In Bantul District, 12 rounds (24 weeks) of egg releases led to *w*Mel being established successfully (Fig 2). The use of adult or egg releases did not influence ultimate success of the deployment. However, communities appeared more accepting of the egg release methodology as it resulted in adult mosquitoes being more gradually introduced into the community as opposed to a pulse of adult mosquitoes being released resulting in a noticeable increase in nuisance biting that was detectable by some community members. Tracking of *Ae*. *aegypti* populations prior and then during and after releases finished indicated that releases did not result in substantially increased mosquito numbers being introduced into communities as measured by BGS trap catches (Fig 2).

In each case the *Wolbachia* frequency estimates approached >80% within six months of releases finishing although there was considerable variation in weekly estimates soon after releases stopped which reduced over time (Fig 2). Long term monitoring of each site by periodic ovitrap sampling (Table 1) confirmed the long-term stability of *w*Mel over a three-year period with frequencies above 80% at all time points measured and > 90% in most estimates.

**Table 1:** Long term monitoring of sites. Sampling was done by ovitrapping periodically in different sites. *Wolbachia* frequency is indicated as a percentage of *Wolbachia* infected individuals sampled. Also indicated are the percentage of *Ae*. *aegypti* positive ovitraps and the total number of mosquitoes sampled to obtain estimate–*Wolbachia* % (% positive traps, total mosquitoes tested).

Site	14 Apr 2016	7 Oct 2016	20 Oct 2016	3 Mar 2017	26 May 2017	15 Sep 2017	16 Nov 2017	15 May 2018	14 Sept 2018
Kronggahan	92.2% (67%, 592)		94.3% (67%, 527)		98.2% (53%, 391)		97.3% (46%, 440)	99.4% (55%, 487)	
Nogotirto (release area only)	83.8% (71%, 358)		95.2% (71%, 274)		82.7% (75%, 353)		98.5% (94%, 273)	87.6% (74%, 299)	
Jomblangan		89.3% (74%, 635)		87.9% (53%, 472)		93.6% (45%, 406)			100% (97%, 589)
Singosaren		97.9% (54%, 240)		98.5% (53%, 471)		94.8% (60%, 538)			97.9% (78%, 714)

There has been recent speculation based on laboratory and semi-field studies that *w*Mel may be an unsuitable *Wolbachia* strain for release in control programs due to high temperatures impacting its maternal inheritance[16]. In concordance with prior releases in Australia [8, 14] the *w*Mel strain actually appears quite stable in this disease endemic tropical setting despite consistently high temperatures (S2 Fig), as is the case in Australia, suggesting that that any effects of high temperatures on maternal transmission are insufficient to impact the high stable frequency of *w*Mel in natural *Ae*. *aegypti* populations in these settings.

To capture community concerns, we established an incident management system at the end of 2013. In 2013–2016 the system collectively recorded 446 reports from residents in the study areas Sleman and Bantul with 31% of them being negative concerns raised by the community (i.e. concern over nuisance biting). Concerns peaked during the release period and then dropped away after releases stopped (S2 Table). The majority of logged feedback from the community was positive in nature and supportive of the program and its objectives.

Overall the community acceptability of *Wolbachia* releases has been very high in all locations where the World Mosquito Program has run field release programs due to the importance and resourcing given to community engagement activities [15]. This was the case in this study as well and even with the quite strict criteria initially imposed by the IRB for individual consent the community was strongly supportive. Clearly individual consent is not a practical way to obtain community authorisation if the project was to run at larger scale and in addition individual consent is not normal practice within Indonesian communities where collective decision making is more common. We found that when we shifted consent to community representative consent then the communities were more comfortable with the process and this approach. In addition, it involved formal community leaders to a greater extent and in turn they were more involved in explaining the project and managing any concerns than in areas where individual consent was obtained. In areas where community consent was obtained there were fewer concerns from community members.

**Fig 3:**
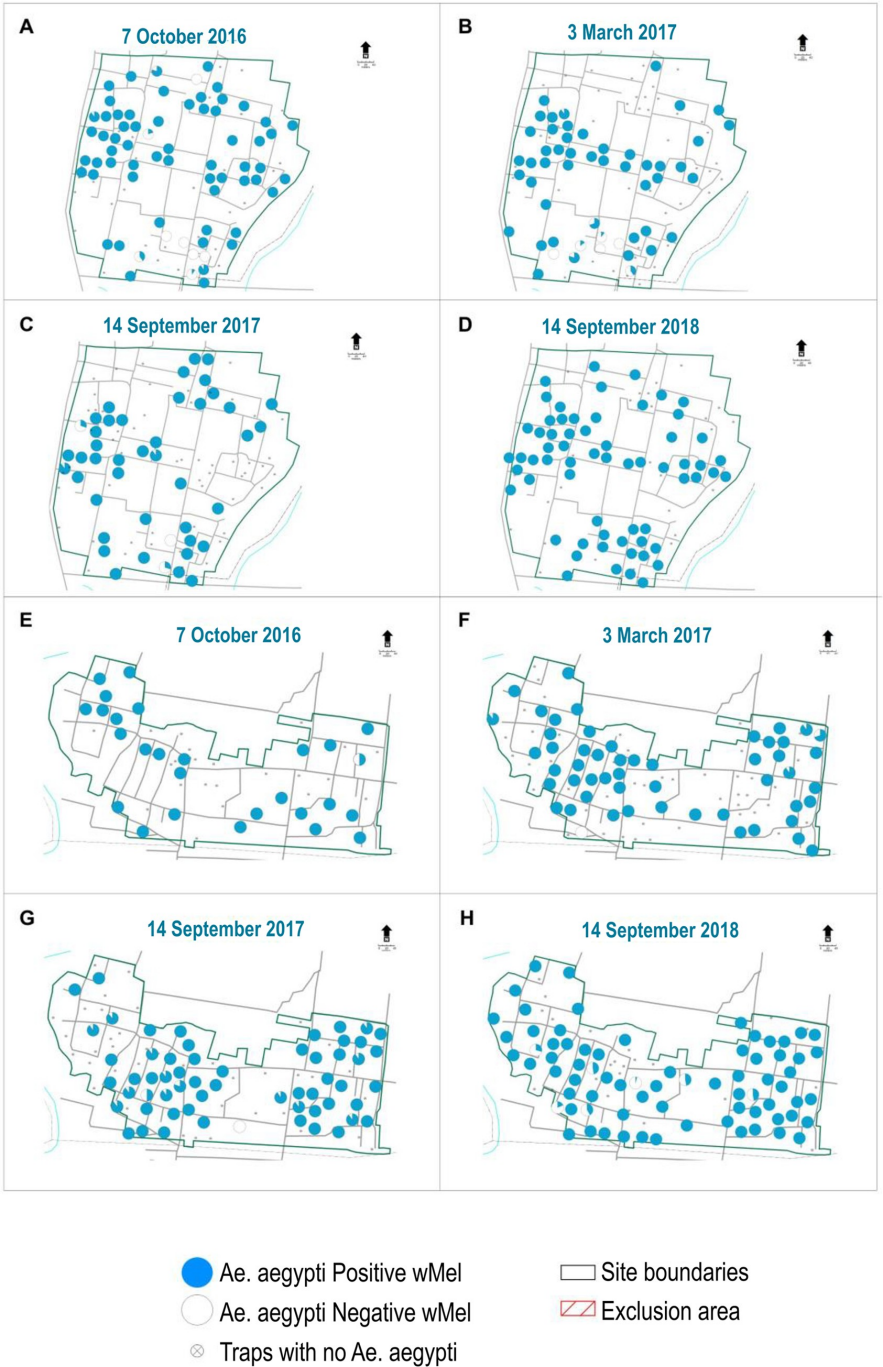
Ovitrap monitoring of *Wolbachia* frequency in Jomblangan (A, B, C, D) and Singosaren (E, F, G, H). Pie graphs show location of where ovitraps were set and the amount of blue shading indicates frequency at each trap from a maximum sample of 10 individuals.

The initial releases in Nogotirto, where individual consent was obtained, were met unusually with community opposition prior to release. This opposition involved approx. 159 people of which around 50% had previously signed individual consent forms, and was organised by a single individual who questioned the safety of the project and questioned issues of compensation for community members if it proved to be unsafe. Out of respect for these concerns and because the community members did not wish to engage in a dialogue around their concerns, we excluded 5 Rukun Tetangga from the release area. This resulted in a section of the Nogotirto field site being treated as an exclusion zone where no releases were undertaken to respect the wishes of the community. This provided a fortuitous control area where it was possible to track the natural spread of the *w*Mel strain into this area (Fig 1). Of note was the slow rate of *w*Mel invasion into this exclusion area over the course of the two-year monitoring period despite wMel being at a consistently high frequency in the adjoining area. While monitoring clearly indicates that the *w*Mel strain invades the exclusion area it does so at a very slow rate, slower than predicted from releases in Australia [10] where *w*Mel spread to a high frequency across several hundred meters across a couple of years. This may reflect overall reduced dispersal behaviour of *Ae*. *aegypti* in these locations compared to Australian sites which may in turn indicate higher habitat suitability in Indonesia. From an operational perspective, the slow rates of spatial spread observed here suggest that natural spreading cannot be relied on to assist deployment programs and that blanket coverage of communities should be considered as the preferred deployment methodology to obtain high rates of coverage in operational deployments. Nonetheless the spread observed here should aid in achieving homogenous coverage across mosquito populations and prevent pockets or “deployment holes” persisting. For completeness ovitrap monitoring data is also provided for Kronggahan (Fig 1) and Jomblangan and Singosaren (Fig 3).

This study has demonstrated that both egg and adult mosquito releases are able to be used successfully to deploy *Wolbachia* reliably and stably in a high dengue transmission setting. Based on these results an egg deployment methodology has been employed for a randomised controlled trial currently running across the city of Yogyakarta [10]. This trial is designed to experimentally measure the reduction in the relative risk of acquiring dengue when *Wolbachia* is established in the mosquito population. These results extend earlier studies in Australia and demonstrate that the methodologies used first in Australia can be extended and used successfully to deploy *Wolbachia* in a disease endemic setting.

## Supporting information

S1 Fig Release sites and proximity to Yogyakarta city. Map supplied by Indonesian Geospatial Information Agency and processed with ArcGIS.
(TIF)

S2 Fig Temperature profile for Yogyakarta Airport during 2013–15. Data supplied by Adisutjipto Climatology Station.
(TIFF)

S1 Table Summary information of four release sites. (DOCX)

S2 Table Summary of community inquiries across release sites 2013–2016 (N = 446). (DOCX)
