# Supplementary figures and images for: Stable establishment of wMel Wolbachia in Aedes aegypti populations in Yogyakarta, Indonesia
Source: PLoS Negl Trop Dis. 2020 Apr 17;14(4):e0008157. doi: 10.1371/journal.pntd.0008157 (PMC7190183; doi:10.1371/journal.pntd.0008157)

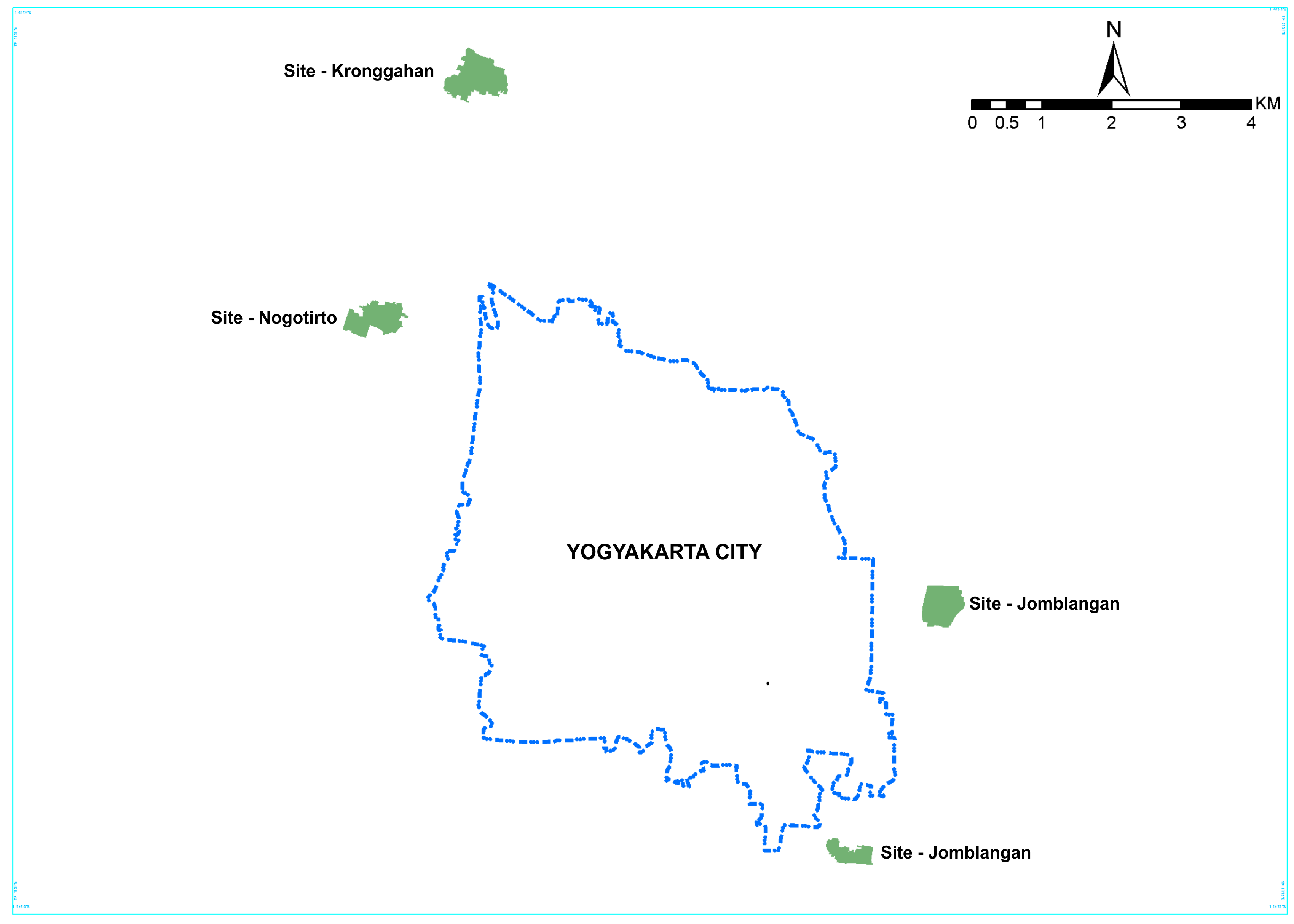

Supplement: S1 Fig — Map supplied by Indonesian Geospatial Information Agency and processed with ArcGIS. (TIF) [file pntd.0008157.s001.tif]

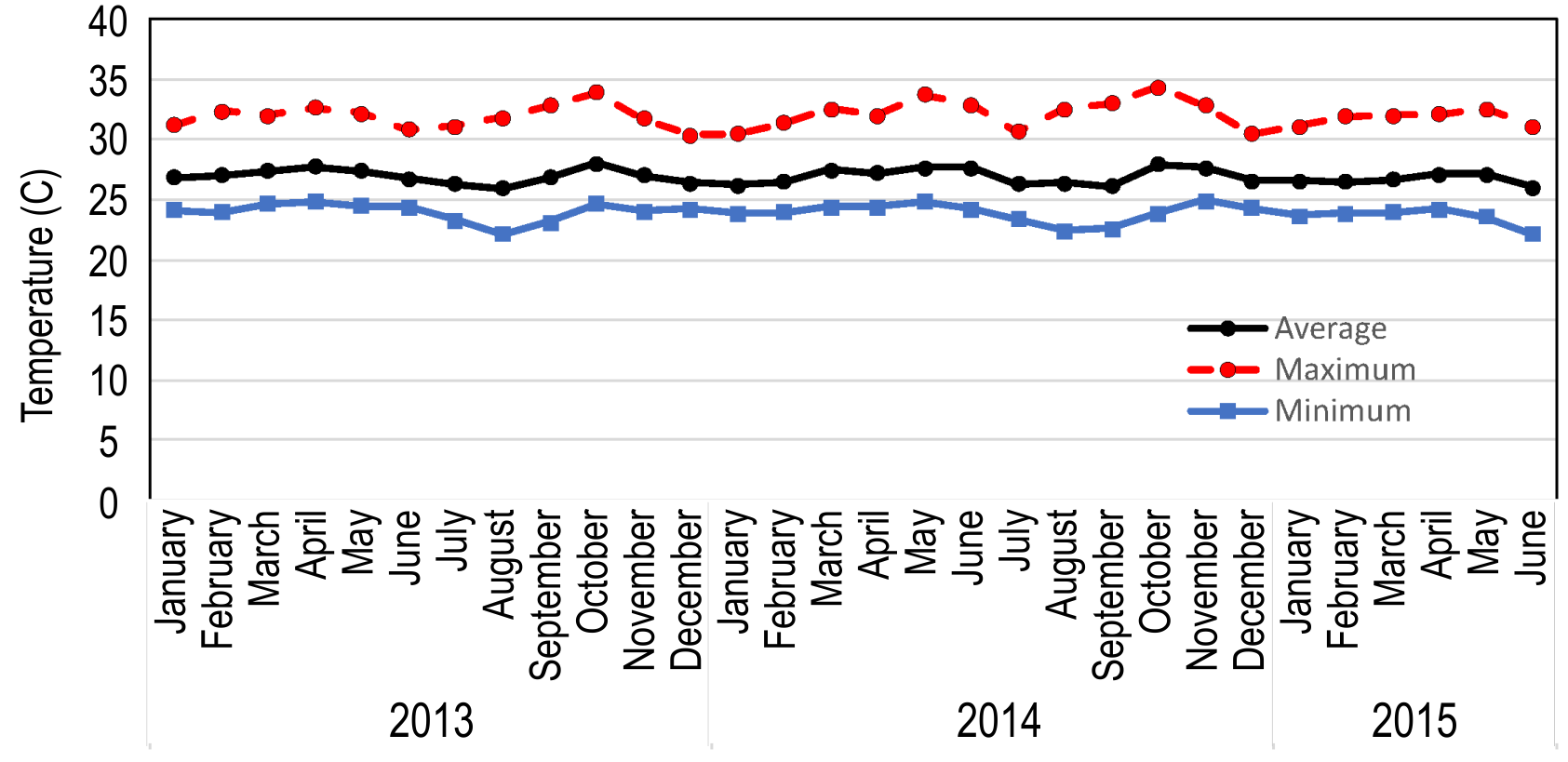

Supplement: S2 Fig — Data supplied by Adisutjipto Climatology Station. (TIFF) [file pntd.0008157.s002.tiff]
