# Supplementary material for: Stable establishment of wMel Wolbachia in Aedes aegypti populations in Yogyakarta, Indonesia
Source: PLoS Negl Trop Dis. 2020 Apr 17;14(4):e0008157. doi: 10.1371/journal.pntd.0008157 (PMC7190183; doi:10.1371/journal.pntd.0008157)
Supplement: S1 Table — (DOCX) [file pntd.0008157.s003.docx]

Table S1. Summary information of four release sites

| Site | Size (km2) | Population | Number of houses | Type of site | Release type |
| --- | --- | --- | --- | --- | --- |
| Jomblangan (Bantul District) | 0.29 | 2530 | 794 | Semi urban | Egg |
| Singosaren (Bantul District) | 0.18 | 1157 | 384 | Urban | Egg |
| Nogotirto (Sleman District) | 0.26 | 2560 | 845 | Semi urban | Adult |
| Kronggahan (Sleman District) | 0.61 | 2681 | 826 | Semi urban | Adult |
