# Supplementary material for: Stable establishment of wMel Wolbachia in Aedes aegypti populations in Yogyakarta, Indonesia
Source: PLoS Negl Trop Dis. 2020 Apr 17;14(4):e0008157. doi: 10.1371/journal.pntd.0008157 (PMC7190183; doi:10.1371/journal.pntd.0008157)
Supplement: S2 Table — (DOCX) [file pntd.0008157.s004.docx]

Table S2. Summary of community inquiries across release sites 2013-2016 (N=446)

|  | Before releases | During releases | 12 month period after releases finished |
| --- | --- | --- | --- |
| Nuisance biting and complaints (negative sentiment) | 10.31% | 19.51% | 1.57% |
| Operational inquiries (eg Trap Fail)(neutral sentiment) | 5.61% | 6.95% | 2.69% |
| Community inquiry (eg request to provide community meeting)(positive sentiment) | 13.45% | 14.57% | 7.17% |
| Report of a dengue case in the area (neutral sentiment) | 1.79% | 13.23% | 3.14% |
|  |  |  |  |
|  | 31.17% | 54.26% | 14.57% |
